# Kidney Function Trajectory within Six Months after Acute Kidney Injury Inpatient Care and Subsequent Adverse Kidney Outcomes: A Retrospective Cohort Study

**DOI:** 10.3390/jpm12101606

**Published:** 2022-09-29

**Authors:** You-Lin Tain, Chien-Liang Liu, Hsiao-Ching Kuo, Chien-Ning Hsu

**Affiliations:** 1 Division of Pediatric Nephrology, Kaohsiung Chang Gung Memorial Hospital, Kaohsiung 833, Taiwan; 2 College of Medicine, Chang Gung University, Taoyuan 333, Taiwan; 3 Department of Industrial Engineering and Management, College of Management, National Yang Ming Chiao Tung University, Hsinchu 300, Taiwan; 4 Department of Pharmacy, Kaohsiung Chang Gung Memorial Hospital, Kaohsiung 833, Taiwan; 5 School of Pharmacy, Kaohsiung Medical University, Kaohsiung 807, Taiwan

**Keywords:** acute kidney injury, kidney function recovery, chronic dialysis, end stage kidney disease, kidney function trajectory

## Abstract

Timing and extent of kidney function recovery after an acute kidney injury (AKI) episode are associated with chronic kidney disease onset and progression. This study aimed to categorize AKI recovery patterns within 6 months after index hospital discharge and associate them with kidney outcomes. This was a retrospective cohort study of 234,867 patients, hospitalized between 2010 and 2017, and classified as AKI or no AKI. Kidney function recovery from pre-hospitalization baseline within 1.5× serum creatinine (SCr) were evaluated at 3 and 6 months after hospital discharge and categorized as persistent non-recovery (PNR: SCr not recovered at 3 and 6 months), non-recovery (NR: SCr not recovered at 6 months), and recovery (SCr recovered at 6 months). A composite of incident chronic kidney disease, kidney replacement therapy, and estimated glomerular filtration rate reduction >30% from baseline and <15 mL/min/1.73 m^2^ was evaluated. Of 14,673 AKI surviving patients, 10.18% had PNR and 14.33% showed NR. Compared with no AKI, PNR and NR of AKI were associated with an increased risk of composite adverse outcomes (adjusted subdistribution hazard ratio (SHR) 4.55; 95% CI, 4.05–5.11; SHR, 3.54; 95% CI, 3.18–3.94, respectively). Patients with NR showed a greater risk of adverse outcomes than those with non-rapid recovery at 3 months after hospital discharge. The AKI recovery pattern within 6 months following inpatient care revealed an increasing continuum of risk of long-term adverse kidney outcomes. Risk stratification and a kidney function monitoring plan at discharge are needed to improve post-AKI care.

## 1. Introduction

The incidence of acute kidney injury (AKI) is rapidly increasing worldwide, with an associated increase in direct medical costs and worsening long-term outcomes [1,2,3,4,5]. The Kidney Disease: Improving Global Outcomes consensus group defines AKI as an increase in the concentration of serum creatinine (SCr) of ≥0.3 mg/dL or ≥150% of the baseline within a 2- or 7-day period, or as a decrease in urine output [6]. Although AKI is reversible, prior study results have suggested that the timing of kidney function recovery after AKI episodes affects chronic kidney disease (CKD) onset, long-term CKD progression, and the survival of patients [7,8,9,10]. Three time-based recovery patterns for AKI (recovery within 2–3 days, within 7 days, or within 90 days) have been recognized to define the extent of kidney function recovery from AKI [10,11,12].

It is important to mention that the extent of kidney function recovery is dependent upon the timing of the baseline kidney function assessment (at admission or prior to the hospitalization for AKI). Most previous AKI recovery studies investigated kidney function recovery from the peak SCr during hospitalization, limited to hospital-acquired AKI (HA-AKI) [7], without investigating AKI at admission (i.e., community-acquired AKI, (CA-AKI)) or lack consideration of baseline kidney function before the AKI hospitalization, which could elucidate variances in the risks of AKI and subsequent AKI consequences. Indeed, although CA-AKI causes less in-hospital mortality than HA-AKI, it has been associated with a higher risk of death than that seen in hospitalized patients with no AKI [13,14,15].

The 22nd Acute Disease Quality Initiative AKI Advisory Group suggested that the initial step toward quality improvement of AKI care starts with primary AKI prevention in an outpatient setting to avoid CA-AKI-associated hospitalization and a continuum of care after hospital discharge post-AKI [16]. Clarifying the subsequent kidney function recovery spectrum after AKI exposure may facilitate the design and development of effective interventions to prevent AKI-CKD, CKD progression, and other sequelae in the target patients. Importantly, the identification of subphenotypes has led to insights into their pathogenesis and the development of personalized approaches to post-AKI care. Thus, we performed an assessment of kidney function trajectories at 3 and 6 months in a cohort of AKI survivors and evaluated whether subsequent recovery patterns of kidney function within 6 months carried modifiable effects on long-term adverse kidney outcomes. We hypothesized that AKI survivors with persistent non-recovery (PNR) or short-term recovery were associated with a higher risk of adverse kidney outcomes, including incident CKD, than those with no AKI.

## 2. Materials and Methods

### 2.1. Data Source

This retrospective cohort study included hospitalized adults of the Acute Kidney Injury Recovery Evaluation Study cohort from the network of Chang Gung Memorial Hospitals in Taiwan. Data from the Chang Gung Research Database (CGRD), an integrated electronic health records dataset that has been described in previous articles [17], were utilized in this study. The CGRD contains the International Classification of Diseases, Ninth/Tenth Revision, Clinical Modification (ICD-9/10-CM) codes, Healthcare Common Procedure Coding System codes, Anatomical Therapeutic Chemical Classification System codes, and laboratory test results of emergency department and in-and-outpatient settings (Supplemental Table S1). Chang Gung Memorial Hospitals (9584 beds) accounted for approximately 11% of Taiwan national health insurance program’s annually covered health services in 2018 [18], including over 9 million emergency and outpatient department visits and 300,000 hospital admissions. The Taiwan national health insurance program covers more than 99% of its 23 million population and has included comprehensive health services since 1997 [19].

### 2.2. Study Cohort

The AKI Recovery Evaluation Study included a cohort of patients aged 20 years or older hospitalized between 1 January 2010 and 31 December 2017 from the network of Chang Gung Memorial Hospitals. Patients who did not have SCr values both at admission and ≤90 days prior to the index hospitalization were first excluded, and then patients who had a kidney transplant or received maintenance dialysis therapy before the index hospitalization, or who were younger than 20 years at the index admission date, were also excluded. 

**Figure 1:**
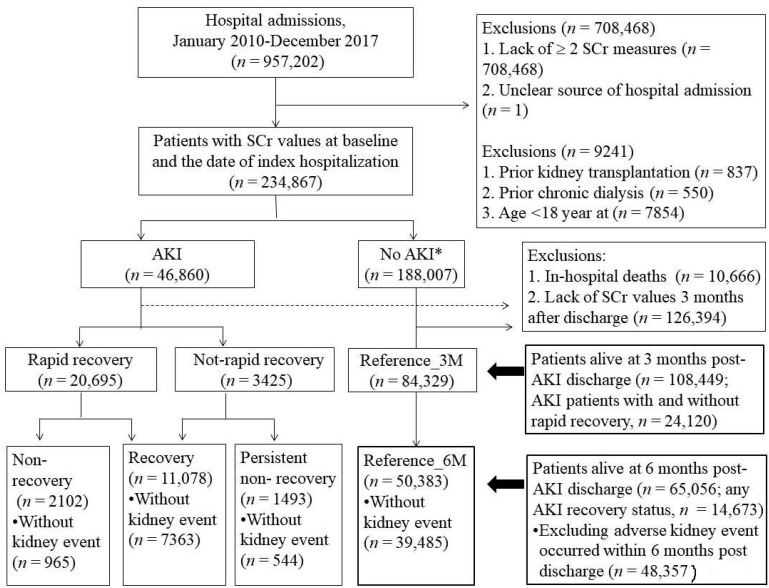
Flow chart of the patient selection AKI: acute kidney injury; not-rapid recovery, defined by the 3-month SCr ≥ 1.5 baseline SCr; * no AKI (neither at admission nor during hospitalization) = control group; non-recovery = post 6-month SCr ≥ 1.5 baseline SCr.

To assess patient kidney function recovery from AKI, hospitalized patients who had data of at least one SCr value both at admission and ≤ 90 days prior to the index hospitalization, survived to 6 months following the index hospital discharge, and had at least one SCr within 3- and 6 months after the discharge were included in this retrospective cohort of Acute Kidney Injury Recovery Evaluation Study (Figure 1). AKI was defined using the modified 2012 Kidney Disease Improving Global Outcomes criteria [6] based on an increase in SCr concentration of at least 50% or 0.3 mg/dL (or more within 2 days) above baseline SCr within 2 and up to 90 days before the index hospital admission [20,21]. Patients with AKI at the index hospital admission were classified as CA-AKI, and the highest SCr concentration during the hospitalization was compared with the index SCr at the admission date to define HA-AKI (i.e., peak SCr > 1.5× SCr at admission). The SCr concentration at or 3 days after the hospital discharge date was considered as the discharge SCr.

### 2.3. Six-Month Kidney Function Trajectory after AKI Hospital Discharge

Our study concept is illustrated in Supplemental Figure S1. AKI recovery was assessed 3 and 6 months after the index hospital discharge as a decrease in SCr < 50% from baseline (full recovery) [22,23]. Rapid recovery and kidney recovery was identified as a <1.5 baseline SCr at 3 months post-AKI discharge (3-month survival cohort) [24]. Further, three distinct patterns of kidney function recovery were assessed at 6 months after the AKI hospital discharge (6-month survival cohort): PNR (SCr not recovered at 3 and 6 months), non-recovery (NR: SCr not recovered at 6 months), and recovery (SCr recovered at 6 months). Patients without AKI during the index hospitalization were considered as the control (reference group) to determine the association between the pattern of AKI recovery and long-term prognosis (Figure 1).

In the primary 6-month survival cohort, the trajectory of kidney function was constructed using data obtained at baseline (3 months prior to the index hospitalization), index admission, hospitalization, discharge, and every 3 months after the hospital discharge to the end of follow-up. Kidney function over the study follow-up was reflected by the estimated glomerular filtration rate (eGFR) using the Taiwan version of Modification of Diet in Renal Disease equation as 175 × SCr (mg/dL)^−1.154^ × age (years)^−0.203^ × 0.742 (if female), with the averaged eGFR value over a 3-month interval [25].

### 2.4. Adverse Kidney Outcomes

Adverse kidney outcomes included the onset of chronic dialysis (determined as any modality of dialysis that continued for at least 3 months, with at least 1 dialysis encounter in each month), kidney transplantation, mean eGFR < 60 mL/min/1.73 m^2^ for ≥3 months (CKD), eGFR reduction ≥30% baseline (last eGFR versus baseline eGFR) [26], and eGFR < 15 mL/min/1.73 m^2^ sustained for at least 2 successive quarters. A composite of adverse kidney outcome was assessed at 6 months after the hospital discharge. The follow-up time was censored at in-hospital death, loss to follow-up, or the latest date of the dataset (31 December 2017), whichever came first. The risk of incident CKD (eGFR < 60 mL/min/1.73 m^2^) sustained for at least 2 successive quarters was assessed in a group of patients without CKD at baseline. To avoid biases, patients who developed any outcome event of interest within 180 days post-index hospital discharge were excluded from further analyses (the adverse kidney outcomes naïve 6-month survival cohort).

### 2.5. Baseline Covariates 

Based on a literature review of factors shown to increase the risk of AKI hospitalization and expert opinions [3], variables of interest included age at the index hospital admission, sex, Charlson Comorbidity Index (CCI) conditions within 1 year before the admission [27], number of outpatient visits, emergency department visits, hospitalizations, and dialysis treatments in the preceding 3 months before index admission were ascertained. Use of intensive care and dialysis during the index hospitalization and timing of AKI occurrence were analyzed. 

### 2.6. Statistical Analysis

Descriptive statistics are presented as mean (SD) or median (interquartile ranges) and frequency (percentage) for continuous and categorical variables, respectively. Variables of interest included the age at the index hospital admission, sex, and Charlson Comorbidity Index (CCI) conditions. The number of outpatient visits, emergency department visits, hospitalizations, dialysis treatments, and classes in the 3 months preceding the index admission were ascertained. The use of intensive care and dialysis during the index hospitalization and the timing of AKI occurrence were also analyzed.

A competing risk of Cox proportional hazards model was employed to determine the independent association of the 6-month kidney recovery pattern and adverse kidney outcomes, controlling for baseline covariates and early death. A sensitivity analysis for the 3-month recovery pattern was performed to elucidate the importance of the follow-up time (3 months versus 6 months) after discharge from AKI hospitalization. Two-sided *p* values < 0.05 were considered statistically significant in all analyses. Data management and statistical analyses were performed using SAS version 9.4 (SAS Institute, Cary, NC, USA).

## 3. Results

### 3.1. Patient Characteristics

Figure 1 illustrates the study analysis flow and comparative groups. A total of 108,449 hospitalized adults [22.24% (*n* = 24,120) of whom were AKI survivors having at least one SCr measurements in 3 months after the index hospital discharge] were included in this study; of that total, 65,056 patients (including 14,673 AKI survivors) remained in the study for the 6-month kidney function recovery assessment (Table 1). Although the primary patient cohort decreased in number, patient characteristics were similar between the 3-month and 6-month cohorts in kidney trajectory analyses (Supplemental Tables S1 and S2). Among the primary 6-month cohort of patients with AKI, 75.5% (*n* = 11,078) showed recovery, 10.2% (*n* = 1493) had PNR, and 14.3% (*n* = 2102) were NR. Excluding 16,999 patients who developed an outcome event within 6 months post-AKI discharge (Supplemental Table S3), the risk of adverse kidney outcomes was evaluated in 48,357 patients with and without CKD at baseline (Table 1).

The patients had a mean age of 59.2 (15.40) years; however, patients with recovery AKI were older (mean age of 63.46 years) than those without AKI and those in the other AKI groups. A larger percentage of patients with recovery AKI and non-AKI were male, and fewer of them had a CCI score > 3 (37.77% vs. 28.34%, respectively) than other AKI groups. Importantly, more patients with PNR- and NR-AKI experienced CA-AKI only (59% vs. 43.4%, respectively) and both CA- and HA-AKI (10.85% vs. 4.04%) than patients with recovery AKI (Table 1). On the other hand, most patients with recovery AKI experienced HA-AKI only (70.31%), and approximately 2% had experienced both CA- and HA-AKI in the index hospitalization.

### 3.2. Kidney Function Trajectory

**Figure 2:**
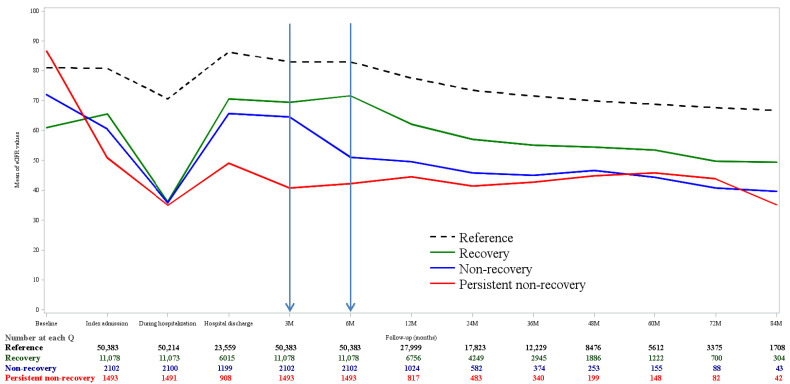
Kidney function trajectories by kidney recovery patterns 6 months after the index hospital discharge (*n* = 65,056). Number of patients indicates patients who had at least one eGFR value in the 3-month window (M = month).

Figure 2 depicts the kidney function (eGFR) trajectories in the pre- and post-AKI follow-up period for AKI and non-AKI patients in the primary 6-month survival cohort (*n* = 65,056). Patients with PNR- or NR-AKI had a higher mean eGFR (86.72 vs. 72.03 mL/min/1.73 m^2^) at baseline than that in those with recovery AKI, but showed a low and sustained eGFR at the 3- (40.70 vs. 64.51 mL/min/1.73 m^2^) and 6-month follow-up (42.15 vs. 51.04 mL/min/1.73 m^2^) (Supplemental Table S4).

### 3.3. Post-AKI Kidney Function Recovery Patterns and Adverse Kidney Outcomes

In the adverse kidney outcomes naïve 6-month survival cohort (*n* = 48,357), the overall unadjusted in-hospital mortality rate was 18.37% (5.9 per 100 person-years, PY); patients with and without CKD showed a similar mortality rate of 6/100 PY following the index hospital discharge (Table 2). Unsurprisingly, the rate of the composite adverse kidney outcomes (including incident CKD) was higher in patients with PNR-AKI (34.26/100 PY), followed by NR- (27.84/100 PY), and recovery AKI (9.51/100 PY) than that observed in patients with non-AKI (8.35/100 PY). The rate of initiation of kidney replacement therapy was 2.5/1000 PY for the entire study cohort and 7/1000 PY for patients with CKD at baseline.

The incident CKD rate was 5.49/100 PY, and it was 2-fold higher in patients with NR AKI (10.34 to 13.16/100 PYS) than in those with recovery AKI and non-AKI (5.22/and 6.22/100 PY, respectively) (Table 2).

**Figure 3:**
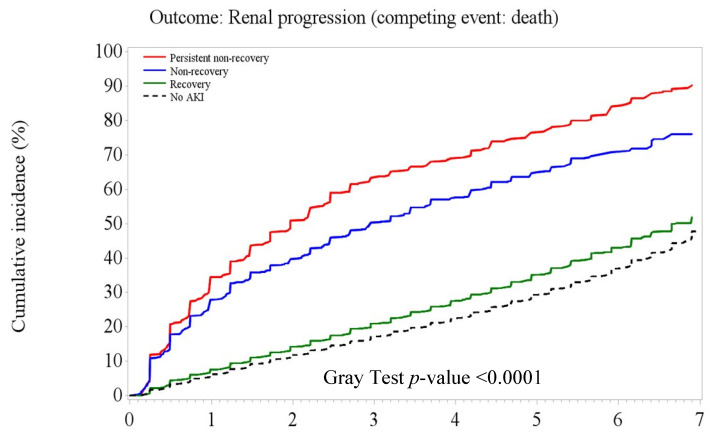
Cumulative risk of adverse kidney outcomes by acute kidney injury recovery patterns at 6 months after the index hospital discharge in the naïve 6-month survival cohort (*n* = 48,357).

**Figure 4:**
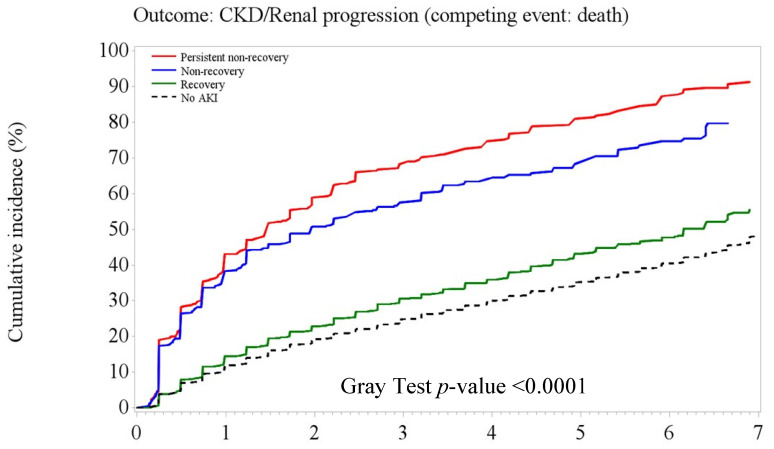
Among patients without prior chronic kidney disease (CKD, *n* = 32,208), the Kaplan–Meier plot shows that the highest risk of composite adverse kidney outcomes (including CKD onset) was in the PNR- and NR-AKI groups, and the risk was lower in the recovery AKI and no-AKI group.

**Figure 5:**
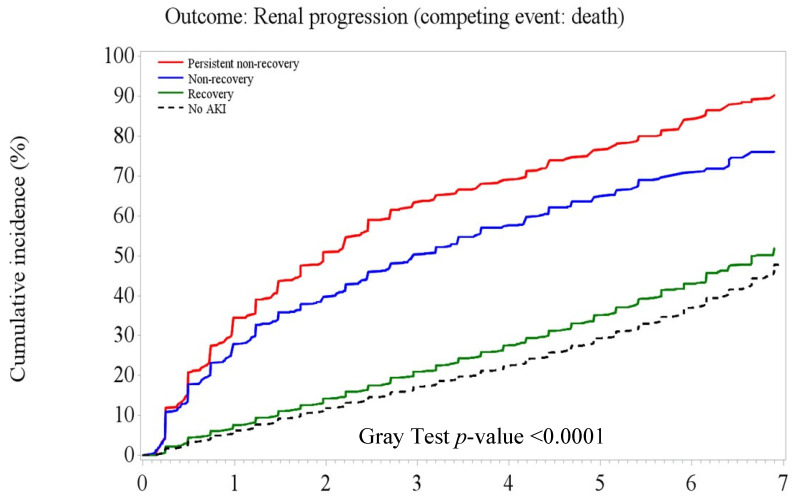
Among patients with prior chronic kidney disease (CKD; *n* = 16,149), the Kaplan–Meier plot shows that the highest risk for composite adverse kidney outcomes was in the PNR- and NR-AKI groups, and the risk was close between the recovery AKI and no-AKI groups. Adverse kidney outcomes: eGFR declined >30% baseline or <15 mL/min/1.73 m^2^, kidney-replacement therapy.

Figure 3, Figure 4 and Figure 5 illustrate the significant difference in the cumulative incidence of adverse kidney outcomes over time in the entire cohort and in those with and without CKD at baseline (Gray tests, all *p* < 0.0001). Overall, patients with PNR- or NR-AKI had substantially higher rates of composite adverse kidney outcomes than patients with recovery AKI and no-AKI over the 7-year follow-up period (Figure 3). Among patients without existing CKD, patients with recovery AKI had a slightly higher cumulative rate of adverse kidney outcomes (including CKD onset) than non-AKI patients (Figure 4). However, the cumulative rates of CKD progression outcomes were close between AKI recovery and non-AKI among those patients with existing CKD (Figure 5). 

AKI recovery pattern-associated with risk of adverse kidney outcomes were consistent in competing risk Cox regression analysis. Compared with patients with non-AKI, the adjusted subdistribution hazard ratio (SHR) indicated that PNR-AKI (SHR, 4.55; 95% CI, 4.05–5.11) was significantly associated with a greater risk of adverse kidney outcomes, and the degree of association was 5-fold higher in patients with CKD at baseline (SHR, 5.38; 95% CI, 4.46–6.49) (Table 3). Compared with non-AKI patients, those with recovery AKI were associated with a 58% higher risk of incident CKD, while patients with PNR- or NR-AKI were associated with a 6-fold higher risk (Table 3). Patient characteristics, uses of healthcare services, and timing of AKI occurrence (as presented in Table 1) were included in the full adjusted regression model.

In the sensitivity analyses, the dose–response association between AKI recovery patterns and adverse kidney outcome risk was consistent between the 3- and 6-months recovery pattern and between those with and without CKD at baseline (Table 3). The risk of adverse kidney outcomes was more sensitive in patients with slow or no AKI at 3 months post-AKI discharge (i.e., patients with PNR AKI).

## 4. Discussion

The present hospitalized cohort study results are novel in AKI literature and demonstrate that kidney function recovery patterns at 6 months after index AKI hospital discharge are associated with long-term adverse kidney outcomes compared with those of hospitalized patients with no AKI. Partial and persistent non-recovery of kidney function in the 6-month AKI cohort after hospital discharge was considered high in the study cohort. Although 86% of the patients experienced a full recovery of kidney function within the first 3 months after the AKI discharge, extending the observation period to 6 months is important for stratifying the risk of sustained non-recovery and short-term recovery with poor long-term kidney outcomes.

AKI non-recovery has been associated with a higher risk of major adverse kidney outcomes; however, duration of non-recovery kidney function and its association with long-term patient outcomes have yet not been systematically studied. In the Alberta healthcare system, 3231 AKI survivors (vs. 880 patients without AKI) were assessed 90 days after discharge (range, 30–150 days); AKI survivors without recovery from kidney function had a four-time higher risk of recurrent AKI (adjusted HR 4.13 (3.38–5.04)) and 26% higher risk of mortality (adjusted HR 1.23 (1.10–1.43)) in the 5-year follow-up [8]. A prospective cohort study (Assessment, Serial Evaluation, and Subsequent Sequelae of Acute Kidney Injury, ASSESS-AKI), including 1538 hospitalized patients with CA-, HA-AKI and no AKI, has reported that patients with non-resolving AKI (72 h post-AKI from the maximum concentration of SCr or returned to the last baseline pre-admission SCr) was significantly associated with a 2.3-fold (95% CI 1.52–3.48) higher risk of major adverse kidney events compared to patients with no AKI [12]. Similar to our study, these studies included pre-hospitalization SCr and evaluated the effect of AKI recovery after 3 months of hospital discharge, but the time frame for AKI recovery was varied within 90 days or 72 h post-AKI event [8,12]. Another study restricted to a cohort of 16,968 critically ill patients with stage 2 or 3 AKI, using the SCr at the hospital admission as baseline, found that patients with relapse, no recovery, or never reversed kidney function at hospital discharge had the worst 1-year survival outcome compared to patients with early sustained kidney function reversal (not meeting AKI stage 1 criteria) [23]. 

This study is one of few, long-term follow-up studies to elaborate kidney function trajectory after an episode of AKI. Previously, the kidney function trajectory in the post-AKI recovery period was primarily based on a hypothetical model. Rather than directly measuring patient outcomes after an episode of AKI, this longitudinal analysis of the hospitalized adults who had repeated SCr measurements in practice further provided us with a better understanding of a full course of AKI recovery from baseline kidney function (pre-hospitalization) and its role in the development of adverse kidney outcomes. Kidney function recovered at 3 months after hospital discharge was suggested to decrease AKI-associated risk of heart failure and all-cause death in the ASSESS-AKI study for evaluating AKI-associated with mortality, and cardiovascular and kidney outcomes among hospitalized patients who survived ≥3 months after discharge [28]. 

In addition, this study extended the observation time frame of AKI recovery from 3 months to 6 months after the index hospital discharge, enabling us to elaborate the effect of slow recovery of AKI on adverse kidney outcomes. The results of the current study showed that 10.2% patients with sustained unrecovered kidney function after AKI discharge (PNR-AKI) was associated with an approximate 5-fold risk of a composite outcome of kidney disease; on the other hand, patients with prior CKD whose kidney function at 6 months recovered to the baseline pre-hospitalization level had a nearly similar risk of composited adverse kidney outcome as patients with no AKI. Although the observed associations need to be validated in further research, these study results supported the need for continuous long-term monitoring for at least 6 months post-AKI to clarify the modifiable prognostic factors of AKI recovery (e.g., kidney function monitoring, nephrontoxin management, or fluid overload avoidance), and thus decrease morbidity and adverse sequelae of AKI as recommended by the Acute Disease Quality Initiative [16]. 

Following the currently recommended AKI criteria [6], this large retrospective cohort study consistently demonstrated that baseline pre-hospitalization SCr and AKI at admission (i.e., CA-AKI) are of the same importance as HA-AKI, resulting in diverse kidney function trajectories. We found that the subset of AKI survivors who experienced more than one episode of AKI during the index hospitalization (CA-AKI, then HA-AKI) were at highest risk among all groups for unlikely recovery by 3 and 6 months after hospital discharge (Supplemental Tables S1 and S2). These study results are coherent with those showing that the kinetics of the eGFR appeared in sharper decline in the 3-month pre-hospital period (eGFR slope between baseline and index admission) in patients with PNR- and NR-AKI (Figure 2). Although the etiology of CA-AKI may differ from HA-AKI, further research to identify risk factors associated with NR-AKI would facilitate the development of a risk-stratified assessment at hospital discharge. 

Prevention of incident CKD and management of CKD progression has been a target in AKI literature. The findings of this study provide valuable insight into the continuity of post-AKI care and complement previously published short-term (≤90 days) kidney recovery trajectory data. Regarding baseline kidney function and multi-comorbidities populations, such as diabetes, glomerular kidney disease, surgeries, or intensive care [4], continuous clinical monitoring for at least 6 months post-AKI discharge could be beneficial to develop predictive models with multi-factorial risk factors to identify patients’ degree of risk during post-AKI follow-up [21,28,29,30]. It is also important for health professionals and patients who experienced AKI to understand the ongoing impacts of AKI on readmission and mortality [31,32,33,34]. 

Recently, KDIGO recommends using the term acute kidney disease (AKD) to cover patients with abnormal kidney function and/or structure with the functional criteria: AKI, eGFR < 60 mL/min/1.73 m^2^, decrease in eGFR by ≥35% or increase in SCr by >50% within 3 months, and/or structural kidney damage (albuminuria, hematuria or pyuria) [35]. The implications of AKD possibly include patients who previously were not CKD (eGFR < 60 mL/min/1.73 m^2^ for longer than 3 months) or patients who were in the post-AKI recovery period and had an eGFR < 60 mL/min/1.73 m^2^. The full spectrum of kidney damage (AKI, AKD, and CKD) is beyond the scope of this AKI recovery evaluation study. However, more research is needed to map these causes and clinical characteristics of AKD patients with and without AKI to long-term major adverse kidney outcomes. 

There are limitations in this study that should be addressed. First, the 6-month AKI recovery patterns after hospital discharge only included patients with a sufficient number of SCr measurements (pre-hospitalization, at hospital admission, and at least one measure within 3 months and at 4–6 months post-hospitalization). A considerable proportion of AKI survivors were excluded due to missing SCr values in the follow-up period, which may limit its generalizability to patients who received a certain degree of medical care. However, this study is unique in that it focuses on the pre-and post-AKI kidney function kinetics and compares them to patients without AKI in the index hospitalization in routine care practice. Second, time-varying residual confounding in the post-AKI follow-up cannot be completely excluded and resulted in the bias toward a higher extent association of patients with PNR-AKI with adverse kidney outcomes. Non-AKI factors, such as non-regular nephrologist follow-up, comorbidities, and nephrotoxins, might accelerate kidney function deterioration. Third, the results may be applicable only in the Taiwanese or populations with universal access to a healthcare system, and may have limited generalizability to the overall population of AKI patients. Last, the duration of AKI dialysis therapy following the index hospital discharge was not assessed, which requires further research to investigate the effect of dialysis therapy on AKI-related outcomes. Nevertheless, the dose-relationship between timing kidney function recovery spectrum and adverse kidney outcomes in the present study could help improve the understanding of risk prevention for AKI-related adverse kidney outcomes. 

## 5. Conclusions

In summary, periodic continuous kidney function assessment and modifiable risk management for at least 6 months after hospitalization of AKI could be beneficial for patients with slow or temporal AKI recovery of kidney function in the real world setting. Patient’s pre-hospital kidney function, etiology of AKI, and time to evaluate eGFR recovered to baseline should be considered in the risk stratification strategy. A better AKI stratification will lead to risk ascertainment on an individual patient basis and ultimately to personalized approaches for AKI care and follow-up. 

## Figures and Tables

**Table 1:** Characteristics of study cohort in the analyses of adverse kidney outcomes.

Overall (*n* = 48,357)		Persistent Non-Recovery (*n* = 544)		Non-Recovery (*n* = 965)		Recovery (*n* = 7363)		No AKI (*n* = 39,485)		*p* Value
Age at index admission, year										<0.001
<65	27,952	342	(62.87)	521	(53.99)	3704	(50.31)	23,385	(59.23)	
≥65	20,405	202	(37.13)	444	(46.01)	3659	(49.69)	16,100	(40.77)	
Sex, *n* (%)										0.01
Male	26,898	275	(50.55)	502	(52.02)	4088	(55.52)	22,033	(55.80)	
Female	21,459	269	(49.45)	463	(47.98)	3275	(44.48)	17,452	(44.20)	
eGFR at baseline, mL/min/1.73 m^2^										
>=60	33,543	376	(69.12)	617	(63.94)	4021	(54.61)	28,529	(72.25)	<0.001
<60	14,814	168	(30.88)	348	(36.06)	3342	(45.39)	10,956	(27.75)	
Dialysis before index admission	24	3	(0.55)	3	(0.31)	13	(0.18)	5	(0.01)	<0.001
Baseline comorbidity										
CCI score										<0.001
0	9002	66	(12.13)	85	(8.81)	870	(11.82)	7981	(20.21)	
1~3	24,701	234	(43.01)	443	(45.91)	3712	(50.41)	20,312	(51.44)	
>3	14,654	244	(44.85)	437	(45.28)	2781	(37.77)	11,192	(28.34)	
Acute myocardial infarction	1086	11	(2.02)	35	(3.63)	278	(3.78)	762	(1.93)	<0.001
Congestive heart failure	2790	45	(8.27)	115	(11.92)	730	(9.91)	1900	(4.81)	<0.001
Peripheral vascular diseases	1049	14	(2.57)	36	(3.73)	261	(3.54)	738	(1.87)	<0.001
Cerebral vascular accident	5268	69	(12.68)	139	(14.40)	1177	(15.99)	3883	(9.83)	<0.001
Dementia	1096	10	(1.84)	36	(3.73)	288	(3.91)	762	(1.93)	<0.001
Pulmonary disease	4636	51	(9.38)	139	(14.40)	915	(12.43)	3531	(8.94)	<0.001
Connective tissue disorder	1063	12	(2.21)	33	(3.42)	208	(2.82)	810	(2.05)	<0.001
Peptic ulcer	7934	96	(17.65)	211	(21.87)	1501	(20.39)	6126	(15.51)	<0.001
Liver diseases	10,256	134	(24.63)	260	(26.94)	1654	(22.46)	8208	(20.79)	<0.001
Diabetes	14,843	180	(33.09)	383	(39.69)	2856	(38.79)	11,424	(28.93)	<0.001
Diabetes complications	4994	53	(9.74)	116	(12.02)	984	(13.36)	3841	(9.73)	<0.001
Paraplegia	565	18	(3.31)	19	(1.97)	150	(2.04)	378	(0.96)	<0.001
Renal disease	6144	109	(20.04)	216	(22.38)	1595	(21.66)	4224	(10.70)	<0.001
Cancer	18,031	236	(43.38)	357	(36.99)	2298	(31.21)	15,140	(38.34)	<0.001
Severe liver diseases	1207	39	(7.17)	80	(8.29)	288	(3.91)	800	(2.03)	<0.001
Metastatic cancer	4996	81	(14.89)	114	(11.81)	633	(8.60)	4168	(10.56)	<0.001
Health service uses during hospitalization										
Dialysis	255	20	(3.68)	23	(2.38)	167	(2.27)	45	(0.11)	<0.001
Intensive care unit	4819	87	(15.99)	165	(17.10)	1164	(15.81)	3403	(8.62)	<0.001
Timing of AKI occurred										
No AKI	39,485	-						39,485		
CA-AKI only (at admission)	2781	321	(59.01)	419	(43.42)	2041	(27.72)	-		
HA-AKI only (during hospitalization)	5848	164	(30.15)	507	(52.54)	5177	(70.31)	-		
CA- and HA-AKI	243	59	(10.85)	39	(4.04)	145	(1.97)	-		
Kidney function recovery within 3 months after discharge, *n* (%)										
Not-rapid recovery	811	544		-		267	(3.63)	-		
Rapid recovery	8061	-		965		7096	(96.37)	-		
Not-rapid recovery	811	544		-		267	(3.63)	-		
Rapid recovery	8061	-		965		7096	(96.37)	-		
Mean (SD) value (*n* = 48,357)										
Age at index admission, year		59.20	(15.40)	62.43	(15.41)	63.46	(15.23)	60.91	(14.13)	<0.001
Baseline eGFR, mL/min/1.73 m^2^		119.62	(67.92)	90.49	(53.29)	70.87	(40.82)	81.11	(33.37)	<0.001
Number of outpatient visit		5.47	(4.61)	5.39	(4.22)	5.31	(4.63)	5.32	(4.12)	0.79
Number of Emergency department visit		0.64	(1.16)	0.72	(1.01)	0.74	(1.12)	0.42	(0.91)	0.001
Number of hospitalizations		0.57	(0.72)	0.54	(0.73)	0.42	(0.65)	0.39	(0.65)	<0.001

- Not applicable; CCI: Charlson comorbidity index; CA-AKI: community-acquired AKI; HA-AKI: hospital-acquired AKI; Baseline eGFR was retrieved < 3 months before the index hospitalization; SD: standard deviation; *p* value for four groups’ comparison; chi-squared test for categorical data and ANOVA for continuous data.

**Table 2:** Adverse kidney outcomes among patients at risk 6 months after index hospital discharge (*n* = 48,357).

	Overall			Persistent Non-Recovery			Non-Recovery			Recovery				No AKI	
	per 100 PY	*n* (%)		per 100 PY	*n* (%)		per 100 PY	*n* (%)		per 100 PY	*n* (%)		per 100 PY	*n* (%)	
Patients at risk, *n*		48,357			544			965			7363			39,485	
Composite adverse kidney outcome (including incident CKD), *n* (%)	9.04	12,415	(25.67)	34.26	435	(79.96)	27.84	563	(58.34)	9.51	1888	(25.64)	8.35	9529	(24.13)
eGFR reduction ³30% baseline	6.56	9754	(20.17)	28.05	422	(77.57)	21.38	511	(52.95)	6.77	1461	(19.84)	5.98	7360	(18.64)
eGFR < 15	1.35	1981	(4.10)	4.63	68	(12.50)	4	94	(9.74)	2.1	444	(6.03)	1.13	1375	(3.48)
Kidney replacement therapy	0.25	377	(0.78)	0.76	12	(2.21)	0.28	7	(0.73)	0.38	82	(1.11)	0.22	276	(0.70)
Chronic dialysis	0.24	362	(0.75)	0.76	12	(2.21)	0.24	6	(0.62)	0.35	76	(1.03)	0.22	268	(0.68)
Kidney transplantation	0.012	18	(0.04)		0		0.041	1	(0.10)	0.027	6	(0.08)	0.01	11	(0.03)
Death	5.9	8881	(18.37)	9.08	145	(26.65)	11.84	293	(30.36)	6.66	1457	(19.79)	5.61	6986	(17.69)
Patients without CKD at baseline, *n*		32,208				355			559			3734			27,580
Composite adverse kidney outcome (including incident CKD) *n* (%)	8.89	7995	(24.82)	34.94	267	(79.70)	28.3	334	(59.75)	10.38	1017	(27.24)	8.16	6377	(23.12)
eGFR reduction ³30% baseline	5.73	5637	(17.50)	28.77	256	(76.42)	20.31	292	(52.24)	6.49	708	(18.96)	5.15	4381	(15.88)
eGFR < 15	0.09	86	(0.27)	0.11	1	(0.30)	0.14	2	(0.36)	0.18	20	(0.54)	0.07	63	(0.23)
Incident CKD	5.48	4968	(15.42)	10.34	84	(25.07)	13.16	160	(28.62)	6.22	614	(16.44)	5.22	4110	(14.90)
Kidney replacement therapy	0.02	24	(0.07)	0	0		0	0		0.02	2	(0.05)	0.03	22	(0.08)
Chronic dialysis	0.02	20	(0.06)	0	0		0	0		0.02	2	(0.05)	0.02	18	(0.07)
Kidney transplantation	0.004	4	(0.01)	0	0		0	0		0	0		0.005	4	(0.01)
Death	5.85	5825	(18.09)	9.19	87	(25.97)	11.46	170	(30.41)	6.53	721	(19.31)	5.63	4847	(17.57)
Patients with CKD at baseline, *n*		16,149				209			406			3629			11,905
Composite adverse kidney outcome *n* (%)	9.33	4420	(27.37)	33.25	168	(80.38)	27.19	229	(56.40)	8.66	871	(24.00)	8.76	3152	(26.48)
eGFR reduction ³30% baseline	8.2	4117	(25.49)	26.995	166	(79.43)	22.99	219	(53.94)	7.05	753	(20.75)	7.84	2979	(25.02)
eGFR < 15	3.97	1895	(11.73)	12.78	67	(32.06)	10.61	92	(22.66)	4.19	424	(11.68)	3.62	1312	(11.02)
Kidney replacement therapy	0.7	353	(2.19)	1.9	12	(5.74)	0.71	7	(1.72)	0.75	80	(2.20)	0.67	254	(2.13)
Chronic dialysis	0.68	342	(2.12)	1.9	12	(5.74)	0.61	6	(1.48)	0.69	74	(2.04)	0.66	250	(2.10)
Kidney transplantation	0.027	14	(0.09)	0	0		0.1	1	(0.25)	0.056	6	(0.17)	0.02	7	(0.06)
Death	5.995	3056	(18.92)	8.93	58	(27.75)	12.41	123	(30.30)	6.8	736	(20.28)	5.56	2139	(17.97)

PY = person-years was calculated from the 6 months post-index hospital discharge to the outcome of interest, death, or censored (whichever came first).

**Table 3:** Associations between kidney recovery patterns and risk of adverse kidney outcomes.

	Overall (*n* = 48,357)		Without CKD (*n* = 32,208) *				With CKD (*n* = 16,149)		
	SHR	95% CI	*p*-Value	SHR	95% CI	*p*-Value	SHR	95% CI	*p*-Value
Composite adverse kidney outcomes *									
**At 6 months**									
Persistent non-recovery	4.55	(4.05–5.11)	<0.001	4.85	(4.20–5.61)	<0.001	5.38	(4.46–6.49)	<0.001
Non-recovery	3.54	(3.18–3.94)	<0.001	3.45	(3.02–3.93)	<0.001	3.73	(3.15–4.41)	<0.001
Recovery	1.30	(1.20–1.40)	<0.001	1.36	(1.24–1.50)	<0.001	1.12	(1.00–1.25)	0.045
**At 3 months**									
Not-rapid recovery	3.85	(3.48–4.26)	<0.001	4.18	(3.69–4.74)	<0.001	4.12	(3.53–4.81)	<0.001
Rapid recovery	1.54	(1.43–1.66)	<0.001	1.63	(1.49–1.79)	<0.001	1.31	(1.18–1.46)	<0.001
**Incident CKD ^#^**									
Persistent/non-recovery				6.23	(5.25–7.39)	<0.001			
Recovery				1.58	(1.39–1.80)	<0.001			

Overall cohort = 48,357 (excluded patients presenting any outcome of interest event within 6 months post-AKI hospital discharge from the primary 6-month survival cohort). * Composite adverse kidney outcomes: last eGFR in the follow-up period reduction ≥30% baseline eGFR, eGFR < 15 mL/min/1.73 m^2^; kidney replacement therapy: chronic dialysis for ≥3 months or kidney transplantation; incident CKD: eGFR < 60 mL/min/1.73 m^2^; ^#^ Incident CKD was included in the sub-cohort patients without CKD at baseline SHR: subdistribution hazard ratio (reference: no AKI) was fully adjusted with patient’s characteristics and health service uses.

## Data Availability

Data are contained within the article.

## Supplementary Materials

The following supporting information can be downloaded at: https://www.mdpi.com/article/10.3390/jpm12101606/s1, Figure S1: Conceptual analytic model of AKI recovery evaluation; Table S1: Characteristics of the patient cohort that survived 3 months after index hospital discharge; Table S2: Characteristics of the study cohort that survived 6 months after index hospital discharge in the kidney trajectory analysis; Table S3: Excluded patients with adverse kidney outcome event within 6 months after index hospital discharge (*n* = 16,699); Table S4: Mean estimated glomerular filtration rates from pre- and post-hospitalization period in the study cohort that survived 6 months after index hospital discharge in the kidney function trajectory analysis (*n* = 65,056).

## References

[1] Li P.K., Burdmann E.A., Mehta R.L. (2013). Acute kidney injury: Global health alert. Intern. Med. J..

[2] Coca S.G., Yusuf B., Shlipak M.G., Garg A.X., Parikh C.R. (2009). Long-term risk of mortality and other adverse outcomes after acute kidney injury: A systematic review and meta-analysis. Am. J. Kidney Dis..

[3] Chertow G.M., Burdick E., Honour M., Bonventre J.V., Bates D.W. (2005). Acute kidney injury, mortality, length of stay, and costs in hospitalized patients. J. Am. Soc. Nephrol..

[4] James M.T., Bhatt M., Pannu N., Tonelli M. (2020). Long-term outcomes of acute kidney injury and strategies for improved care. Nat. Rev. Nephrol..

[5] Heung M., Steffick D.E., Zivin K., Gillespie B.W., Banerjee T., Hsu C.Y., Powe N.R., Pavkov M.E., Williams D.E., Saran R. (2016). Acute Kidney Injury Recovery Pattern and Subsequent Risk of CKD: An Analysis of Veterans Health Administration Data. Am. J. Kidney Dis..

[6] Kellum J.A., Lameire N., Aspelin P., Barsoum R.S., Burdmann E.A., Goldstein S.L., Herzog C.A., Joannidis M., Kribben A., Levey A.S. (2012). Kidney Disease: Improving Global Outcomes (KDIGO) Acute Kidney Injury Work Group KDIGO clinical practice guidelines for acute kidney injury. Kidney Int. Suppl..

[7] Macedo E., Mehta R.L. (2014). Targeting recovery from acute kidney injury: Incidence and prevalence of recovery. Nephron Clin. Pract..

[8] Pannu N., James M., Hemmelgarn B., Klarenbach S. (2013). Association between AKI, recovery of renal function, and long-term outcomes after hospital discharge. Clin. J. Am. Soc. Nephrol..

[9] Sawhney S., Marks A., Fluck N., Levin A., Prescott G., Black C. (2017). Intermediate and long-term outcomes of survivors of acute kidney injury episodes: A large population-based cohort study. Am. J. Kidney Dis..

[10] Siew E.D., Abdel-Kader K., Perkins A.M., Greevy R.A., Parr S.K., Horner J., Vincz A.J., Denton J., Wilson O.D., Hung A.M. (2020). Timing of Recovery From Moderate to Severe AKI and the Risk for Future Loss of Kidney Function. Am. J. Kidney Dis..

[11] Chawla L.S., Bellomo R., Bihorac A., Goldstein S.L., Siew E.D., Bagshaw S.M., Bittleman D., Cruz D., Endre Z., Fitzgerald R.L. (2017). Acute kidney disease and renal recovery: Consensus report of the Acute Disease Quality Initiative (ADQI) 16 Workgroup. Nat. Rev. Nephrol..

[12] Bhatraju P.K., Zelnick L.R., Chinchilli V.M., Moledina D.G., Coca S.G., Parikh C.R., Garg A.X., Hsu C.Y., Go A.S., Liu K.D. (2020). Association between early recovery of kidney function after acute kidney injury and long-term clinical outcomes. JAMA Netw. Open.

[13] Hsu C.N., Lee C.T., Su C.H., Wang Y.C., Chen H.L., Chuang J.H., Tain Y.L. (2016). Incidence, Outcomes, and Risk Factors of Community-Acquired and Hospital-Acquired Acute Kidney Injury: A Retrospective Cohort Study. Medicine.

[14] Wonnacott A., Meran S., Amphlett B., Talabani B., Phillips A. (2014). Epidemiology and outcomes in community-acquired versus hospital-acquired AKI. Clin. J. Am. Soc. Nephrol..

[15] Soto K., Campos P., Pinto I., Rodrigues B., Frade F., Papoila A.L., Devarajan P. (2016). The risk of chronic kidney disease and mortality are increased after community-acquired acute kidney injury. Kidney Int..

[16] Kashani K., Rosner M.H., Haase M., Lewington A.J.P., O’Donoghue D.J., Wilson F.P., Nadim M.K., Silver S.A., Zarbock A., Ostermann M. (2019). Quality Improvement Goals for Acute Kidney Injury. Clin. J. Am. Soc. Nephrol..

[17] Shao S.C., Chan Y.Y., Kao Yang Y.H., Lin S.J., Hung M.J., Chien R.N., Lai C.C., Lai E.C. (2019). The Chang Gung Research Database-A multi-institutional electronic medical records database for real-world epidemiological studies in Taiwan. Pharmacoepidemiol. Drug Saf..

[18] National Health Insurance Administration 2018 Annual Report of Health Services Claims, by Health Care Organizations. https://www.nhi.gov.tw/Content_List.aspx?n=8A5CA04F618E3364&topn=23C660CAACAA159D.

[19] Cheng S.H., Chiang T.L. (1997). The effect of universal health insurance on health care utilization in Taiwan: Results from a natural experiment. JAMA.

[20] Hsu C.N., Liu C.L., Tain Y.L., Kuo C.Y., Lin Y.C. (2020). Machine Learning Model for Risk Prediction of Community-Acquired Acute Kidney Injury Hospitalization From Electronic Health Records: Development and Validation Study. J. Med. Internet Res..

[21] Liu C.L., Tain Y.L., Lin Y.C., Hsu C.N. (2022). Prediction and Clinically Important Factors of Acute Kidney Injury Non-recovery. Front. Med..

[22] Bellomo R., Ronco C., Kellum J.A., Mehta R.L., Palevsky P. (2004). Acute renal failure—Definition, outcome measures, animal models, fluid therapy and information technology needs: The Second International Consensus Conference of the Acute Dialysis Quality Initiative (ADQI) Group. Crit. Care.

[23] Kellum J.A., Sileanu F.E., Bihorac A., Hoste E.A.J., Chawla L.S. (2017). Recovery after Acute Kidney Injury. Am. J. Respir. Crit. Care Med..

[24] Vanmassenhove J., Vanholder R., Lameire N. (2018). Points of concern in post acute kidney injury management. Nephron.

[25] Chen L.I., Guh J.Y., Wu K.D., Chen Y.M., Kuo M.C., Hwang S.J., Chen T.H., Chen H.C. (2014). Modification of diet in renal disease (MDRD) study and CKD epidemiology collaboration (CKD-EPI) equations for Taiwanese adults. PLoS ONE.

[26] Coresh J., Turin T.C., Matsushita K., Sang Y., Ballew S.H., Appel L.J., Arima H., Chadban S.J., Cirillo M., Djurdjev O. (2014). Decline in estimated glomerular filtration rate and subsequent risk of end-stage renal disease and mortality. JAMA.

[27] Sundararajan V., Henderson T., Perry C., Muggivan A., Quan H., Ghali W.A. (2004). New ICD-10 version of the Charlson comorbidity index predicted in-hospital mortality. J. Clin. Epidemiol..

[28] Ikizler T.A., Parikh C.R., Himmelfarb J., Chinchilli V.M., Liu K.D., Coca S.G., Garg A.X., Hsu C.Y., Siew E.D., Wurfel M.M. (2021). A prospective cohort study of acute kidney injury and kidney outcomes, cardiovascular events, and death. Kidney Int..

[29] Lee B.J., Hsu C.Y., Parikh R., McCulloch C.E., Tan T.C., Liu K.D., Hsu R.K., Pravoverov L., Zheng S., Go A.S. (2019). Predicting Renal Recovery After Dialysis-Requiring Acute Kidney Injury. Kidney Int. Rep..

[30] James M.T., Pannu N., Hemmelgarn B.R., Austin P.C., Tan Z., McArthur E., Manns B.J., Tonelli M., Wald R., Quinn R.R. (2017). Derivation and External Validation of Prediction Models for Advanced Chronic Kidney Disease Following Acute Kidney Injury. JAMA.

[31] Siew E.D., Parr S.K., Wild M.G., Levea S.L., Mehta K.G., Umeukeje E.M., Silver S.A., Ikizler T.A., Cavanaugh K.L. (2019). Kidney disease awareness and knowledge among survivors ofacute kidney injury. Am. J. Nephrol..

[32] Silver S.A., Harel Z., McArthur E., Nash D.M., Acedillo R., Kitchlu A., Garg A.X., Chertow G.M., Bell C.M., Wald R. (2017). 30-day readmissions after an acute kidney injury hospitalization. Am. J. Med..

[33] Sawhney S., Marks A., Fluck N., McLernon D.J., Prescott G.J., Black C. (2017). Acute kidney injury as an independent risk factor for unplanned 90-day hospital readmissions. BMC Nephrol..

[34] Silver S.A., Goldstein S.J., Harel Z., Harvey A., Rompies E.J., Adhikari N.K., Acedillo R., Jain A.K., Richardson R., Chan C.T. (2015). Ambulatory care after acute kidney injury: An opportunity to improve patient outcomes. Can. J. Kidney Health Dis..

[35] Lameire N.H., Levin A., Kellum J.A., Cheung M., Jadoul M., Winkelmayer W.C., Stevens P.E., Caskey F.J., Farmer C.K., Fuentes A.F. (2021). Harmonizing acute and chronic kidney disease definition and classification: Report of a Kidney Disease: Improving Global Outcomes (KDIGO) Consensus Conference. Kidney Int..

