# Supplementary material for: Kidney Function Trajectory within Six Months after Acute Kidney Injury Inpatient Care and Subsequent Adverse Kidney Outcomes: A Retrospective Cohort Study
Source: J Pers Med. 2022 Sep 29;12(10):1606. doi: 10.3390/jpm12101606 (PMC9605305; doi:10.3390/jpm12101606)
Supplement: Supplementary file 1 [file jpm-12-01606-s001.zip › jpm-1798666-supplementary.pdf]

Supplementary Materials

Supplemental Figure S1: Conceptual analytic model of AKI recovery evaluation

Supplemental Table S1. Characteristics of the patient cohort that survived 3 months after index hospital discharge

Supplemental Table S2. Characteristics of the study cohort that survived 6 months after index hospital discharge in the kidney trajectory analysis

Supplemental Table S3. Excluded patients with adverse kidney outcome event within 6 months after index hospital discharge (n=16,699)

Supplemental Table S4. Mean estimated glomerular filtration rates from pre- and post-hospitalization period in the study cohort that survived 6 months after index hospital discharge in the kidney function trajectory analysis (n=65,056)

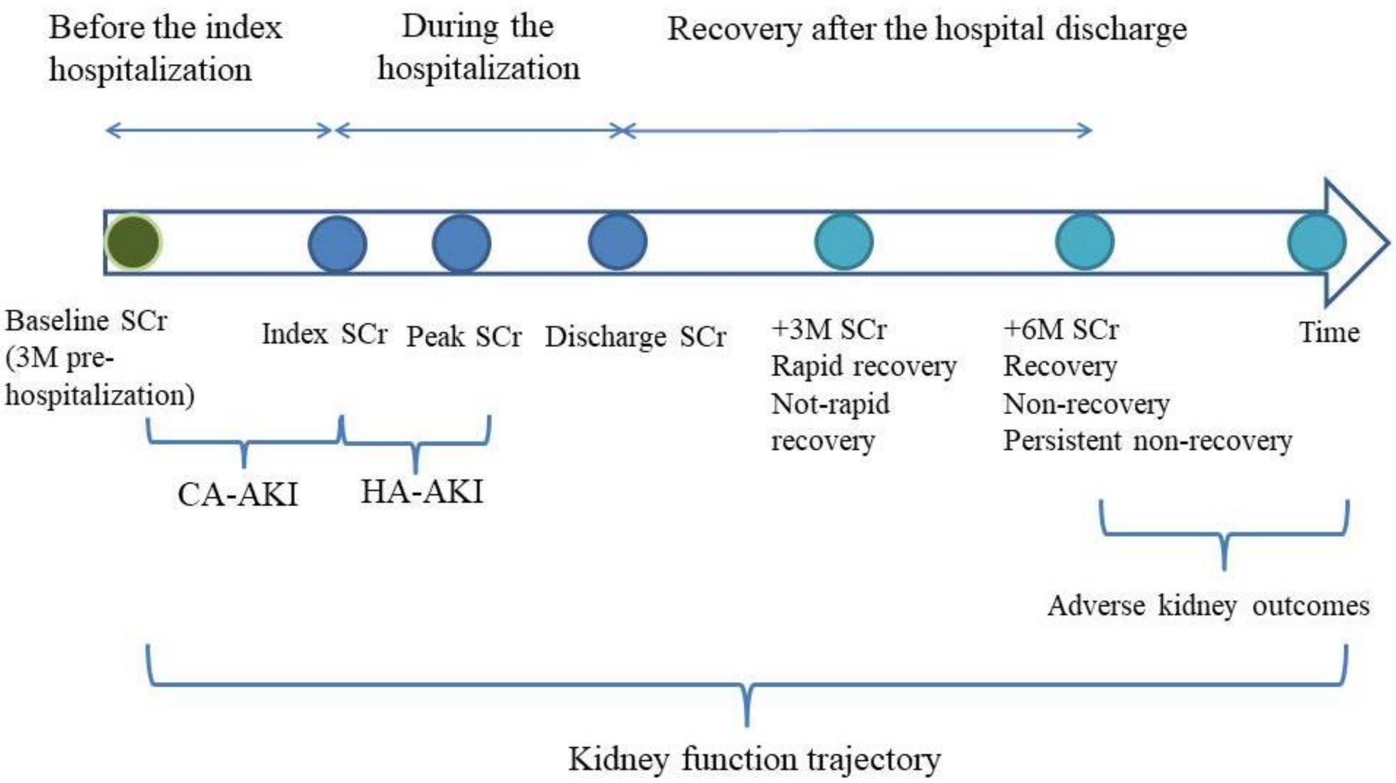

Supplemental Figure S1: Conceptual analytic model of AKI recovery evaluation after 3- and 6-month from acute kidney injury hospital discharge. SCr: serum creatinine; CA-AKI: community-acquired acute kidney injury; HA-AKI: hospital-acquired acute kidney injury; Rapid recovery (+3M SCr<1.5×baseline SCr); Recovery AKI: +6M SCr <1.5×baseline SCr)

Supplemental Table S1: Characteristics of the patient cohort that survived 3 months after index hospital discharge

	Overall (n=1,08,449)	Not-rapid recovery (n=3,425)	Rapid recovery (n=20,695)	No AKI (n=84,329)	*p* value
Age at index admission, years, n (%)					<.001
<65	59578	1738 (50.74)	9740 (47.06)	48100 (57.04)	
≥65	48871	1687 (49.26)	10955 (52.94)	36229 (42.96)	
Sex, n (%)					<.001
Male	60705	1799 (52.53)	11378 (54.98)	47528 (56.36)	
Female	47744	1626 (47.47)	9317 (45.02)	36801 (43.64)	
Dialysis before index admission, n (%)	246	37 (1.08)	199 (0.96)	10 (0.01)	<.001
Baseline comorbidity, n (%)					
CCI group					<.001
0	24707	408 (11.91)	2737 (13.23)	21562 (25.57)	
1~3	53156	1473 (43.01)	9999 (48.32)	41684 (49.43)	
>3	30586	1544 (45.08)	7959 (38.46)	21083 (25.00)	
AKI occurred, n (%)					-
No AKI	84329	-	-	84329	
CA-AKI only (at admission)	9509	2030 (59.27)	7479 (36.14)	-	
HA-AKI only (during hospitalization)	13775	1075 (31.39)	12700 (61.37)	-	
CA- and HA-AKI	836	320 (9.34)	516 (2.49)	-	
Health service uses during hospitalization					
Dialysis	2303	364 (10.63)	1806 (8.73)	133 (0.16)	<.001
Intensive care unit	11888	513 (14.98)	3324 (16.06)	8051 (9.55)	<.001
**Mean (SD)**					
Age at index admission, year	108449	63.23 (15.30)	64.59 (15.40)	61.34 (14.94)	<.001
Baseline eGFR, ml/min/1.73m^2	108449	85.90 (59.13)	64.02 (45.13)	81.28 (33.15)	<.001
Number of outpatient visit	108449	5.48 (4.83)	5.68 (6.18)	5.08 (4.10)	<.001
Number of emergency department visit	108449	0.77 (1.10)	0.80 (1.18)	0.48 (0.92)	<.001
Number of hospitalization	108449	0.54 (0.72)	0.45 (0.65)	0.40 (0.64)	<.001

CCI: Charlson comorbid index; CA-AKI: community-acquired AKI; HA-AKI: hospital-acquired AKI;-Baseline eGFR, number of outpatient visit, emergency department and hospitalization were retrieved <3months before the index hospitalization; SD: standard deviation; *p* value for three groups' comparison: Chi-squared test for categorical data and ANOVA for continuous data.

Supplemental Table S2: Characteristics of the study cohort that survived 6 months after index hospital discharge in the kidney function trajectory analysis

	Overall (n=65,056)	Persistent non-recovery (n=1,493)	Non-recovery (n=2,102)	Recovery (n=11,078)	No AKI (n=50,383)	*P* value
Age at index date, years, n (%)						<0.001
<65	36812	836 (55.99)	1026 (48.81)	5481 (49.48)	29469 (58.49)	
≥65	28244	657 (44.01)	1076 (51.19)	5597 (50.52)	20914 (41.51)	
Sex, n (%)						<0.001
Male	36088	752 (50.37)	1138 (54.14)	5986 (54.04)	28212 (56.00)	
Female	28968	741 (49.63)	964 (45.86)	5092 (45.96)	22171 (44.00)	
Dialysis before index date, n (%)	132	22 (1.47)	12 (0.57)	92 (0.83)	6 (0.01)	<0.001
Baseline eGFR, ml/min/1.73m^2, n (%)						<.0001
≥60	44113	887 (59.41)	1106 (52.62)	5237 (47.70)	36883 (73.21)	
<60	20943	606 (40.59)	996 (47.38)	5841 (52.73)	13500 (26.79)	
Baseline comorbidity, n (%)						
CCI score						<0.001
0	11905	160 (10.72)	163 (7.75)	1191 (10.75)	10391 (20.62)	
1~3	32319	633 (42.40)	878 (41.77)	5420 (48.93)	25388 (50.39)	
>3	20832	700 (46.89)	1061 (50.48)	4467 (40.32)	14604 (28.99)	
**During hospitalization, n (%)**						
In-hospital dialysis	1504	194 (12.99)	181 (8.61)	1060 (9.57)	69 (0.14)	<0.001
Intensive care	6575	232 (15.54)	322 (15.32)	1645 (14.85)	4376 (8.69)	<0.001
AKI occurred, n (%)						
CA-AKI only (at admission)	6014	957 (64.10)	1027 (48.86)	4030 (36.38)	-	
HA-AKI only (during hospitalization)	8153	377 (25.25)	987 (46.96)	6789 (61.28)	-	
CA- and HA-AKI	506	159 (10.65)	88 (4.19)	259 (2.34)	-	
**Recovery at 3 months after index hospital discharge, n (%)**						
Not-rapid recovery	1911	1493	-	418 (3.77)	-	
Rapid recovery	12762	-	2102	10660 (96.23)	-	
**Mean (SD)**						
Age at index hospital admission	65056	61.47 (14.85)	64.34 (15.03)	63.82 (15.03)	61.12 (14.27)	<0.001
Baseline eGFR	65056	86.72 (63.94)	72.03 (54.38)	60.93 (43.81)	81.02 (33.50)	<0.001
Sum number of prior medications classes	65056	2.18 (1.62)	2.31 (1.59)	2.29 (1.59)	1.78 (1.50)	<.0001
Analgesics (NSAID, Opioids)	19392	421 (28.20)	607 (28.88)	3690 (33.31)	14674 (29.12)	<.0001
Antimicrobial	16244	469 (31.41)	640 (30.45)	3584 (32.35)	11551 (22.93)	<.0001
Antiepileptics	429	14 (0.94)	13 (0.62)	102 (0.92)	300 (0.60)	0.0009
Antihypertension (RASi/diuretics)	20344	601 (40.25)	901 (42.86)	4384 (39.57)	14458 (28.70)	<.0001
Constrast media	4726	72 (4.82)	68 (3.24)	502 (4.53)	4084 (8.11)	<.0001
Antihypeglycemia	15855	395 (26.46)	644 (30.64)	3206 (28.94)	11610 (23.04)	<.0001
Calcineurin inhibitors /	4636	150 (10.05)	154 (7.33)	784 (7.08)	3548 (7.04)	0.0002

methotrexate						
Uric acid lowering agent	4573	136 (9.11)	255 (12.13)	1310 (11.83)	2872 (5.70)	<.0001
Anti-inflammation/intestine	569	4 (0.27)	7 (0.33)	91 (0.82)	467 (0.93)	0.0015
Antihistamines/ Antipsychotics/ Antispasmodics	13523	373 (24.98)	579 (27.55)	2884 (26.03)	9687 (19.23)	<.0001
Bisphosphonates	264	3 (0.20)	9 (0.43)	56 (0.51)	196 (0.39)	0.1991
Digoxin	937	25 (1.67)	54 (2.57)	218 (1.97)	640 (1.27)	<.0001
Statins/fibrates	11740	283 (18.96)	420 (19.98)	2373 (21.42)	8664 (17.20)	<.0001
Lithium	44	0 (0.00)	2 (0.10)	12 (0.11)	30 (0.06)	0.2170
Nitrates	4277	166 (11.12)	254 (12.08)	1081 (9.76)	2776 (5.51)	<.0001
Anticoagulants (oral and heparin)	5751	145 (9.71)	239 (11.37)	1097 (9.90)	4270 (8.48)	<.0001

CCI: Charlson comorbid index; CA-AKI: community-acquired AKI; HA-AKI: hospital-acquired AKI; NSAIDs: nonsteroidal antiinflammatory drug; RASi: Renin-angiotensin-aldosterone system inhibitor;Baseline eGFR, number of medical encounters (outpatient visit, emergency department, and hospitalization), 16 pharmacological classes of medications for drug-induced kidney disease were retrieved <3months before the index hospitalization; SD: standard deviation; *p* value for three groups' comparison: Chi-squared test for categorical data and ANOVA for continuous data

Supplemental Table S3. Excluded patients with adverse kidney outcomes within 6 months after index hospital discharge (n=16,699)

	Overall (n=16,699)	Persistent non-recovery (n=949)	Non-recovery (n=1,137)	Recovery (n=3,715)	No AKI (n=10,898)
Patient excluded, % of 6M cohort	25.67%	45.15%	54.09%	33.53%	21.63%
Baseline eGFR, ml/min/1.73m2, n (%)					
≥60	10570	511 (53.85)	489 (43.01)	1216 (32.73)	8354 (76.66)
<60	6129	438 (46.15)	648 (56.99)	2499 (67.27)	2544 (23.34)
Outcomes of interest, n (%)					
eGFR reduction >30% baseline	3829	825 (86.93)	564 (49.60)	517 (13.92)	1923 (17.65)
eGFR <15 ml/min/1.73m^2	3001	393 (41.41)	429 (37.73)	1722 (46.35)	457 (4.19)
Kidney replacement therapy	1399	151 (15.91)	154 (13.54)	795 (21.40)	299 (2.74)
Incident CKD	2211	405 (42.68)	135 (11.87)	168 (4.52)	1503 (13.79)

To avoid bias, 16,699 patients who developed events of interests ≤ 180 days were excluded from further analyses for the risk of incident adverse kidney outcomes.

Supplemental Table S4. Mean estimated glomerular filtration rates from pre- and post-hospitalization period in the study cohort that survived 6 months after index hospital discharge in the kidney function trajectory analysis (n=65,056)

6-month	Baseline	Index admission	During Hospitalization	Discharge	Post-3M	Post-6M	Post-12M	Post-24M	Post-36M	Post-48M	Post-60M	Post-72M	Post-84M
Persistent non-recovery (n=1493)	86.72	50.83	34.99	49.02	40.70	42.15	44.54	41.35	42.75	44.89	45.87	43.77	35.14
Non-Recovery (n=2102)	72.03	60.42	35.74	65.68	64.51	51.04	49.59	45.87	45.01	46.59	44.37	40.78	39.63
Recovery (n=11078)	60.93	65.56	36.05	70.51	69.48	71.60	62.07	57.00	55.07	54.39	53.46	49.71	49.30
No AKI (n=50383)	81.02	80.68	70.54	86.36	82.92	82.90	77.52	73.40	71.47	69.84	68.73	67.63	66.67

M= months; The number in the table is mean eGFR (ml/min/1.73m^2) same as Figure 2 presents the kidney function trajectory.
